# Orchestrated regulation of immune inflammation with cell therapy in pediatric acute liver injury

**DOI:** 10.3389/fimmu.2023.1194588

**Published:** 2023-06-22

**Authors:** Mingyue Duan, Xiaoguai Liu, Ying Yang, Yanmin Zhang, Rongqian Wu, Yi Lv, Hong Lei

**Affiliations:** ^1^ Department of Clinical Laboratory, The Affiliated Children’s Hospital of Xi’an Jiaotong University, Xi’an, China; ^2^ Key Laboratory of Precision Medicine to Pediatric Diseases of Shaanxi Province, Shaanxi Institute for Pediatric Diseases, The Affiliated Children’s Hospital of Xi’an Jiaotong University, Xi’an, China; ^3^ Department of Infectious Diseases, The Affiliated Children’s Hospital of Xi’an Jiaotong University, Xi’an, China; ^4^ National Local Joint Engineering Research Center for Precision Surgery and Regenerative Medicine, The First Affiliated Hospital of Xi’an Jiaotong University, Xi’an, China

**Keywords:** acute liver injury, inflammation, damage associated molecular patterns, macrophages, cell therapy

## Abstract

Acute liver injury (ALI) in children, which commonly leads to acute liver failure (ALF) with the need for liver transplantation, is a devastating life-threatening condition. As the orchestrated regulation of immune hemostasis in the liver is essential for resolving excess inflammation and promoting liver repair in a timely manner, in this study we focused on the immune inflammation and regulation with the functional involvement of both innate and adaptive immune cells in acute liver injury progression. In the context of the severe acute respiratory syndrome coronavirus-2 (SARS-CoV-2) pandemic, it was also important to incorporate insights from the immunological perspective for the hepatic involvement with SARS-CoV-2 infection, as well as the acute severe hepatitis of unknown origin in children since it was first reported in March 2022. Furthermore, molecular crosstalk between immune cells concerning the roles of damage-associated molecular patterns (DAMPs) in triggering immune responses through different signaling pathways plays an essential role in the process of liver injury. In addition, we also focused on DAMPs such as high mobility group box 1 (HMGB1) and cold-inducible RNA-binding protein (CIRP), as well as on macrophage mitochondrial DNA-cyclic GMP-AMP synthase (cGAS)-stimulator of interferon genes (STING) signaling pathway in liver injury. Our review also highlighted novel therapeutic approaches targeting molecular and cellular crosstalk and cell-based therapy, providing a future outlook for the treatment of acute liver injury.

## 1 Introduction

Acute liver injury (ALI) is characterized by a rapid decline of hepatic function with serum aminotransferases rising from mild to substantial levels, jaundice, and impaired coagulation function. It manifests in patients with baseline liver diseases and identifiable causes of liver damage, or without preexisting liver disease. Severe acute liver injury for fewer than 26 weeks duration with encephalopathy and an international normalized ratio (INR) higher than 1.5 in a patient without cirrhosis or preexisting liver disease is grouped into acute liver failure, which is a devastating life-threatening condition with a mortality of 5-10% (1). Usually, mild and moderate liver injury results in rapid and efficient regeneration through hepatocyte proliferation, whereas severe acute liver injury leads to failure of regeneration and involves a high risk of progression to acute liver failure (2). Pediatric acute liver failure (PALF) differs from adult acute liver failure due to the type and diversity of causes, and the subset of children with indeterminate etiology comprises up to 50% of the PALF population. Compared with groups of definite etiology, children with ALF of indeterminate etiology were more likely to require liver transplantation.

Upon appropriate immune activation by pathogens or tissue damage in the liver, tightly regulated inflammation is a homeostatic inflammatory process that resolves inflammation and promotes tissue regeneration, thus avoiding pathological consequences. Conversely, excessive and dysregulated hepatic immune and overactive inflammation in the liver lead to dyshomeostasis and pathological conditions. As the largest organ in the human body, the liver contains not only liver sinusoidal endothelial cells (LSECs), hepatocytes, hepatic stellate cells (HSCs), but also diverse populations of resident immune cells, such as Kupffer cells (KCs), dendritic cells (DCs), T cells, natural killer (NK) cells, natural killer T (NKT) cells, etc., which play central roles in hepatic immune balance. Therefore, the orchestrated regulation of immune hemostasis in the liver is essential for resolving excess inflammation and promoting liver repair and regeneration in a timely manner (3–5).

This review, therefore, focused on the immune activation and regulation with molecular and cellular crosstalk in the liver during acute liver injury/failure. Based on that, therapeutic strategies and targets for acute liver injury/failure have been summarized, which may provide clues to new therapeutic investigations on acute liver injury, especially in children (6).

## 2 Pediatric acute liver injury/failure in the SARS-CoV-2 pandemic era

Causes of acute liver injury/failure are diverse, including mainly para-acetaminophen (APAP) toxicity, cholestatic liver injury (CLI), alcoholic liver disease (ALD), non-alcoholic steatohepatitis (NASH), hepatic ischemia-reperfusion injury (I/R), virus-related liver injury, and hepatitis of unknown reasons in children (7, 8). Cause-based treatment of pediatric acute liver failure has been comprehensively reviewed by Deep et al. (1). However, virus-related liver injury became another important issue, especially in the SARS-CoV-2 pandemic era (9–11). Becchetti et al. reported alterations in liver enzymes among liver-transplanted patients with SARS-CoV-2 infection and increased in-hospital fatality in a European cohort of liver transplant recipients (12). New evidence for liver injury from SARS-CoV-2 infection has also been provided (13). As the primary receptor for SARS-CoV-2 cellular entry, the angiotensin-converting enzyme 2 (ACE2) receptors, which are expressed not only in the lung parenchyma but also in the gastrointestinal tract and liver epithelia, function in directing the contribution of the virus to the hepatic injury (11). Over-activated immune responses also contribute to liver injury in patients with SARS-CoV-2 infection, leading to cytokine storm syndrome, which may cause acute hepatitis and acute liver failure, even multiple organ failure (14–16).

Acute severe hepatitis of unknown origin in children is another new important issue that has gained international concern since it was first reported in Scotland on 31 March 2022. From 1 October 2021 to 8 July 2022, a total of 1,010 probable cases fulfilling the WHO case definition have been reported from 35 countries, of which 46 (5%) have required liver transplantation (LT), and 22 (2%) died. The etiology of this severe acute hepatitis in children is still not clear and under investigation. Based on the WHO working case definition, laboratory testing has excluded hepatitis A-E viruses that are known to cause acute viral hepatitis. Among the 1,010 probable cases, many cases had PCR assays for pathogen detection such as human adenovirus (HAdV) and SARS-CoV-2. According to the available data, HAdV was the most frequently detected pathogen with a cumulative of 209 PCR-positive cases (21%), and SARS-CoV-2 was detected in 78 cases (8%). In the European region, SARS-CoV-2 was detected by PCR in 16% of cases (54/335) and HAdV in 52% of cases (193/368). In the UK, SARS-CoV-2 was detected in 17% of cases (34/196) while HAdV was detected in 66% of cases (142/216). In the United States of America, SARS-CoV-2 was detected in 8% of cases (15/197). Reports from Japan indicated that SARS-CoV-2 was detected in 8% of cases (5/59) while HAdV was detected in 9% of cases (5/58) (17). A UK study reported that during the 2022 outbreak, over 50% of children with acute hepatitis without liver failure, and all indeterminate pediatric acute liver failure (ID-PALF) cases (10/10) had a presentation of adenoviremia, whereas the proportion of adenovirus-associated ID-PALF to all ID-PALF cases was lower from 2017 to 2021 (3/6, 1/4, 2/3, 0/3, and 0/5, respectively). The presentation of adenoviremia was probably linked to the higher rates of adenovirus infection in children during the outbreak, revealing that the 2022 outbreak was not a newly emerging disease (18). A retrospective review in the US showed an almost twofold increase of non-A-E severe acute hepatitis in children during 2021-2022 compared with the period of 2018-2021, which was associated with higher positive rates for viruses, with adenovirus (26.1%) and SARS-CoV2 (10.3%) being the most frequently detected viruses during the outbreak (19).

The results so far revealed that acute severe hepatitis in children may be related to HAdV or SARS-CoV-2 infection. It seems that HAdV infection was a facilitatory factor rather than a primary driver in the causation of the 2022 outbreak. A proposed mechanism is that superantigen-mediated aberrant immune response to HAdV in the gut following SARS-CoV-2 infection results in excessive IFN-γ release and thus in IFN- γ-mediated hepatocyte apoptosis (20, 21). SARS-CoV-2 virus in the gastrointestinal tract may release viral proteins across the intestinal epithelium and thereby accelerate the immune activation afterward. The superantigen hypothesis is so far the most popular theory to explain multisystem inflammatory syndrome in children (20, 21). Within the SARS-CoV-2 spike protein, there is a superantigen motif that resembles staphylococcal enterotoxin B. This superantigen can trigger strong and broad non-specific T cell activation, which was supported by the expansion of TRBV11-2 T cells in multisystem inflammatory syndrome in children (22–24). In addition to autoimmune hepatitis-like liver histology with CD8^+^ lymphocyte-predominant infiltration, Th1-type immune skewing, with remarkable peripheral CD8^+^ T-cell activation, was found in children with severe acute hepatitis of unknown origin (25).

## 3 Immune activation in acute liver injury/failure

**Figure 1:**
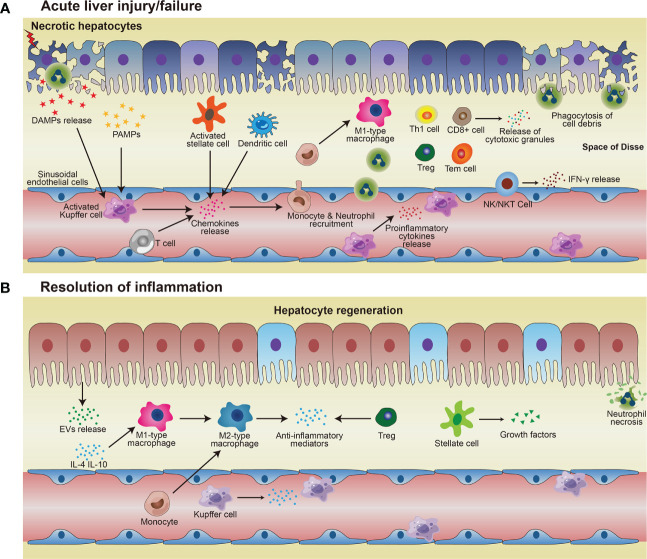
Immune responses in acute liver injury/failure and regeneration. **(A)** KCs and MoMF recognize DAMPs and PAMPs and then being activated, thus releasing pro-inflammatory cytokines to enhance inflammation and damage the local tissue. Activated KCs and other cells such as T cells, stellate cells, dendritic cells, and hepatocytes, secrete chemokines and thus attract monocytes and neutrophils to the site of injury. Monocytes migrate across the liver’s sinusoidal endothelial cell layer and differentiate into M1-type macrophages which exacerbate injury by pro-inflammatory cytokines. Neutrophils and macrophages phagocytose necrotic debris and apoptotic bodies. **(B)** In the process of resolution, restorative M2 macrophages phenotype release anti-inflammatory cytokines and promote neutrophil apoptosis, resulting in homeostasis restoration in the liver. Hepatocytes proliferate to replenish the lost parenchyma. Treg also contributes to anti-inflammatory conditions, to facilitate hepatocyte regeneration.

Regulation of immune homeostasis within the liver has attracted much attention, especially under several pathological conditions. From the innate immunological aspect, the monocyte-macrophage system mediates liver inflammation and plays an essential role in acute liver injury and liver failure (26–28). Macrophages in the liver are mainly KCs, most of which are intravascular to engulf toxic solutes in blood, forming a powerful phagocyte network and providing efficient immune surveillance in the liver. Some KCs are also found in perivascular space interacting with HSCs and hepatocytes. On one hand, KCs combat foreign invaders such as bacteria and viruses; on the other hand, these cells contribute to the immune-compromised microenvironment in the liver to harness antigenic stimuli such as metabolic products by suppressing MHC expression in HSCs and hepatocytes (29, 30). Upon activation, KCs and monocyte-derived macrophages (MoMF) release pro-inflammatory cytokines to exacerbate inflammation and cause tissue damage. Neutrophils and macrophages begin to phagocytose necrotic debris and apoptotic bodies. Activated Kupffer cells, dendritic cells, stellate cells, and T cells facilitate the recruitment of neutrophils and monocytes from the systemic circulation *via* the secretion of chemoattractants such as CCL2 and IL-17 (31–33) (**Table 1**). Additionally, there are also NK cells, NKT cells, and γð T cells, which all contribute to the immune surveillance in the liver (55). The activated NK and NKT cells play roles in liver injury by impairing liver regeneration *via* TNF-α and IFN-γ release (53). γδ T-cells are also involved in the activation of neutrophils in liver fibrosis (54) (**Figure 1A**).

**Table 1:** The mediators (cytokines/chemokines/others) from various immune cells in liver inflammation during the injury/repair process.

Cell type	Released mediators	Main functions
Macrophage	M1-like: IL-6, TNF-α, IL-1β, IL-12, IL-18 CCL2, CXCL9, CXCL10, CXCL11, CCL15, CCL20 M2-like: TGF-β, IL-10	➢ release various proinflammatory cytokines thereby enhancing inflammation and activating other immune cells, and recruit other immune cells, propagating a Th1 type immune response. ➢ perform anti-inflammatory functions for resolution of inflammation and subsequent tissue repair by clearing cells debris (34–52)
Liver-infiltrating monocyte	TNF-α, IL-6, IL-1β, IFN-γ, IL-8 CCL1, CCL2, CCL3, CCL5 IL-10, TGF-β, G-CSF, GM-CSF	➢ recruited *via* receptor CCR2 ➢ secrete proinflammatory cytokines to aggravate inflammation, augment the recruitment of inflammatory cells ➢ involved in the resolution of inflammation and wound healing (31–33)
KCs	IL-6, TNF-α, IL-1β, IL-18 CCL2, CCL5, CXCL1, CXCL2, CXCL8, CXCL16 IL-10, TGF-β	Amongst the main cytokine producers to enhance the inflammation and tissue damage, recruit monocytes and neutrophils, advance hepatocyte necrosis, and mediate resolution and induce proliferation of surviving hepatocytes (29, 30)
Hepatocyte	TNF-α, DAMPs, free radicals CCL2, CCL3, CCL5, CXCL9, CXCL10 TGF-β	➢ damaged hepatocytes release stress signals such as TNF-α and free radicals ➢ activate resting KCs and HSCs ➢ secrete chemotactic mediators for formation of an inflammatory infiltration (20, 21, 29, 30)
NK/NKT cells	IFN-γ, TNF-α	promote liver injury and impair the liver regeneration (53)
γδ T cells	IL-17, IFN-γ	involved in the activation of neutrophils to enhance inflammation (54)
Treg	IL-13	promote macrophage engulfment of apoptotic cells *via* Vav1-Rac1 signaling pathway (48)
iNKT	IL-4	promote hepatocyte proliferation and macrophage transition to pro-restorative phenotype (40)
DCs	IL-6, TNF-α, CCL2	depletion of DCs leads to exacerbated liver injury (35)
CD8^+^ T cells	TNF-α, Granzyme, Perforin	activate death pathways of sensitized hepatocytes and induce necrosis (41–44)

Following injury, proper resolution of the inflammation is the prerequisite for tissue repair and regeneration. Monocyte-macrophage system and cells, such as regulatory T cells (Treg) and HSCs, essentially mediate the resolution of inflammation. Induced by released anti-inflammatory mediators, such as IL-10 and TGF-β, from the inflamed liver (56), more pro-inflammatory M1-type macrophages could be polarized into restorative M2-type macrophages (34), which release anti-inflammatory cytokines and promote angiogenesis and neutrophil apoptosis, therefore aiding homeostasis restoration and tissue recovery. Depletion of DCs resulted in aggravation of liver injury, with a significantly higher expression of IL-6, CCL2, and TNF-α in serum, suggesting a protective role of DCs in liver injury (35). The mediators (cytokines/chemokines/etc.) from various immune cells in liver inflammation and repair processes are summarized in **Table 1** (36, 37). Cells with immune suppressive function such as Treg, *via* facilitating osteoblast differentiation (38), and myeloid-derived suppressor cells (MDSCs), by promoting angiogenesis (46), are other important players within liver regeneration. Hepatocytes proliferate to replenish the lost parenchyma (**Figure 1B**). Self-antigen-driven invariant NKT (iNKT) cells promote hepatocyte proliferation and macrophage transition to pro-restorative phenotype *via* IL-4, thereby facilitating liver regeneration (40).

Besides the aforementioned, many studies revealed evidence that T cells played a role as central regulators in dysregulated and hyper-inflammatory immune activation in the liver, especially in PALF. As PALF is a complex and rapidly evolving clinical syndrome and almost 50% of PALF cases received the diagnosis of indeterminate without specific etiology, there are emerging insights into immune-mediated pathophysiology in PALF, revealing the role of T cells in inflammatory dysregulation in PALF. A model based on the percent of perforin and granzyme expression on CD8^+^ lymphocytes, absolute count of CD8^+^ T cells, and sIL2R predicted a high activation of circulating lymphocytes and linked to indeterminate etiology of PALF (41, 42). The characteristic phenotype of indeterminate PALF intrahepatic immune cells was perforin^+^CD103^+^CD8^+^ T-cell, which performs effector functions and expresses surface markers of tissue-resident memory T cells (43, 44). Other studies also reported that indeterminate PALF cases were characterized by predominant CD8^+^ T-cell infiltrates in the liver tissue (45, 46). Treg, following injections of CCl_4_, were remarkably expanded, and depletion of Treg enhanced the liver inflammation, demonstrating that Treg controls liver inflammation *via* regulating the aberrant activation and functions of immune effector cells (47). A mechanistic study expanded the role of Treg cells in inflammation resolution by secreting IL-13, which stimulates IL-10 production in macrophages, thus promoting macrophage engulfment of apoptotic cells *via* the Vav1-Rac1 signaling pathway (48). A transcriptional analysis identified an immune-driven PALF group, in which over 90% of patients were indeterminate PALF cases with moderate to dense CD8 staining, expressing increased levels of gene signatures for adaptive immune cells including Th1 cells, regulatory T cells, T effector memory cells, cytotoxic T cells, and innate immune cells including macrophages and activated dendritic cells. In addition, gene signatures for several immune cells, for instance, T central memory cells, Th2 cells, Th17 cells, and B cells, were not found significantly expressed in the indeterminate PALF group (49, 50). Although the exact mechanisms have not been fully clarified yet, the determined high levels of a gene signature of T cells, such as CD8^+^ T cells, Th1 cells, and T effector memory cells, indicate that a complex immune network regulates immune‐mediated liver injury in indeterminate PALF.

Extracellular vesicles (EV) were also reported to contribute to the M2 macrophage polarization (51, 52, 57). In a CCL_4_-induced ALI mouse model, the effects of EVs released from hepatocytes and other non-parenchymal cells on hepatic macrophages were explored, indicating that EVs play a pivotal role in liver regeneration by depolarizing MoMF and inducing Kuffer cells to M2-type (58, 59). Although M2-like macrophage response promotes injured liver repair and regeneration, the immune suppressive mediators released into the circulation can be harmful and lead to immune-compromised status, therefore increasing the susceptibility to infections, even immune paresis in sepsis in PALF (60–62). Therefore, immune evaluation for children with indeterminate, progressive hepatitis or indeterminate ALF should be carried out, including assessments for T cell activation, macrophage activation, NK cell function, and other serum biochemical markers (63). Such precise immune monitoring and evaluation will help to understand the phases of liver injury and the complicated coordination between immune cells and liver cells during the regeneration phase.

## 4 Molecular crosstalk between immune cells in the liver during acute liver injury/failure

Behind the cellular communication of immune cells in the liver, molecular crosstalk between them is essential for maintaining the “gatekeeper” function of hepatic immunity. During liver injury, release and activation of DAMPs result in a wide range of immune responses, including KCs activation, neutrophil recruitment to the site of injury, and induction of proinflammatory cytokines such as TNF-α that activate the NF-κB pathway in the hepatocyte (64).

**Figure 2:**
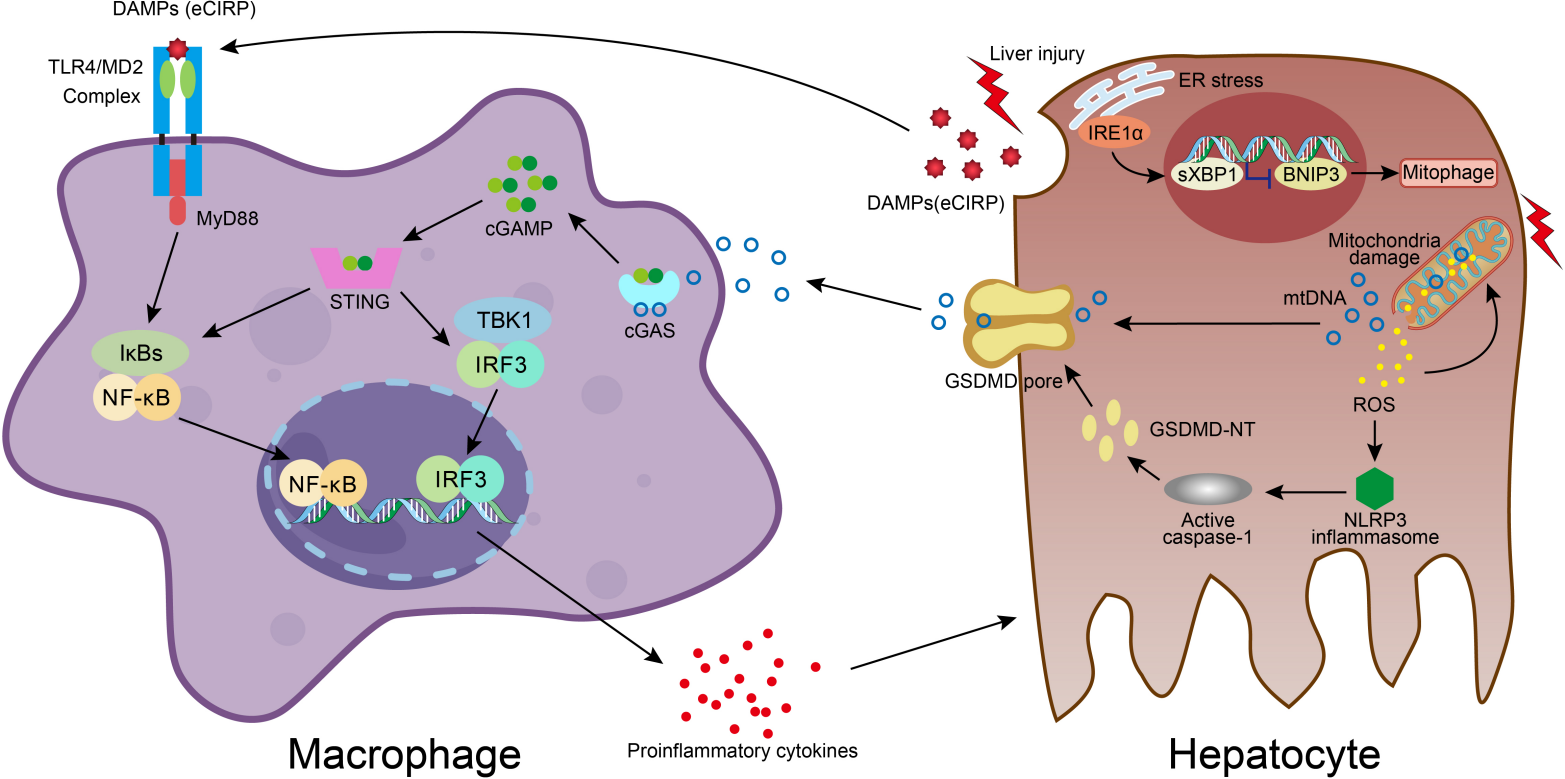
Molecular crosstalk between immune cells and liver cells in acute liver injury. DAMPs molecule eCIRP, released from damaged hepatocyte, stimulates macrophage through TLR4/MD2 complex and NF-κB pathway, resulting in proinflammatory mediators release, which exacerbates hepatocyte injury. In hepatocyte, accumulation of ROS in dysfunctional mitochondria upon damage leads to ROS-NLRP3-caspase-1-GSDMD activation and hepatocyte pyroptosis, which facilitates mtDNA extracellular release. Macrophage engulfs mtDNA and then the cGAS/cGAMP/STING signaling is activated with the secretion of proinflammatory cytokines. The liver injury also activates the IRE1a-XBP1 in the ER stress signaling pathway and leads to BNIP3-mediated mitophagy, which regulates mitochondrial homeostasis and promotes the clearance of cytosolic mtDNA. The XBP1 deficiency promotes mitophagy and thus effects the mtDNA release, and subsequently reduces the STING activation in macrophage.

High mobility group box 1 (HMGB1) is one of the most characterized DAMPs. HMGB1/TLR-4/NF-ĸB signaling directs the triggering of inflammation, innate and adaptive immune responses, and tissue healing after damage. Experimental evidence indicates that HMGB1 release from necrotic hepatocytes seems to be critical for neutrophils and monocyte recruitment, injury exacerbation, and lethality in paracetamol-mediated liver injury (65). Cold shock protein such as cold-inducible RNA-binding protein (CIRP) is a new DAMP molecule participating in acute live injury/failure and sepsis. Extracellular CIRP mainly stimulates monocytes/macrophages through TLR4/myeloid differentiation factor 2 (MD2) complex and the NF-κB pathway, promoting inflammatory responses (**Figure 2**) (66–69). On the other hand, the superfamily of heat shock proteins is also signaling molecules involved in the inflammatory response and restoration of homeostasis. Inhibition of HSP90 reduces proinflammatory cytokine production and prevents LPS-induced liver injury (70), whereas induction of endogenous HSP70 and HSP27 or treatment with exogenous HSP70 ameliorates the hepatic injury during experimental septic shock (71–73). HSPA12A, an atypical member of the heat shock protein 70 family, attenuates LPS-induced liver injury by inhibiting hepatocyte pyroptosis *via* PGC-1α-mediated AOAH expression (74). IL-33, also a classic hepatic DAMPs molecule, is actively involved in various inflammatory pathologies (75, 76). IL-6 trans-signaling drives the coagulopathy and hepatic endotheliopathy associated with COVID-19, which could be a possible mechanism behind liver injury as well (77).

As hepatocyte cell death is a major event and responsible for disease progression in acute liver injury, we also summarized different modes of cell death in immune dyshomeostasis. It is believed that DAMPs are released into the extracellular space largely in necrosis and necroptosis modes due to the loss of plasma membrane integrity and eventually cellular rupture, whereas integral plasma membrane and apoptotic bodies of apoptotic cells constrain DAMPs from being released into extracellular space. X-box binding protein 1 (XBP1) modulates the macrophage proinflammatory response under several pathogenic conditions. While activation of mitochondrial DNA (mtDNA)-cyclic GMP-AMP synthase (cGAS)-stimulator of interferon genes (STING) signaling in macrophage plays an important role in acute liver injury, it also plays a role in the activation of XBP1-mediated hepatocellular mitophagy and pyroptosis. Macrophage STING signaling pathways were observed in human livers with ALI and also acute lung injury in mice (**Figure 2**) (78–81). Pyroptosis is a profoundly inflammatory mode of regulated cell death related to the innate immune system. It has evolved to remove intracellular pathogens and has a cell-bursting morphology associated with pores in the plasma membrane formed by activated Gasdermin D (GSDMD) (82). It has been reported that inhibition and mutation of TNF-α could suppress the effects of HMGB1, thereby inhibiting the process of pyroptosis. The TNF-α/HMGB1 inflammation signaling pathway plays an important role in pyroptosis during liver failure and acute kidney injury (AKI) (83). Ferroptosis is also involved in APAP-induced cell death in primary hepatocytes, and Ferrostatin-1 as a ferroptosis inhibitor plays a protective role in APAP-treated primary hepatocytes (84).

## 5 Therapeutic strategies and targets for acute liver injury

Liver transplantation is the only curative treatment currently available for PALF, but there is a scarcity of livers for donation, thus clinical needs are not met. Novel therapy approaches have been explored that are less invasive than liver transplantation and could reduce posttransplant rejection risks and long-term immunosuppression management. As DAMPs play a pivotal role in the development and progression of acute liver disease, DAMPs can be harnessed as therapeutic targets. Targeting molecules such as HMGB1, HSP, CIRP, circulating free DNA, S100 proteins, and extracellular histones *via* reducing their release, promoting their removal, or inhibiting their signaling could be a promising strategy to ameliorate liver inflammation and minimize organ damage. For instance, treatment with a partly humanized anti-HMGB1 monoclonal antibody has hepatoprotective effects due to it blocking excessive amounts of extracellular HMGB1s, which decreases serum levels of ALT and microRNA-122 and eliminates inflammatory mediators in APAP-induced ALI (85). Accumulating evidence has shown that inhibition of different cell death modes can be utilized as a therapeutic strategy for acute liver diseases. Administration of ferric chelator Deferoxamine (Desferal) after APAP overdose significantly delayed the development of APAP-induced hepatotoxicity in mice (86). The synthetic compound Fer-1, which scavenges initiating alkoxyl radicals produced by ferrous iron from lipid hydroperoxides to produce the same anti-ferroptotic effect as GPx4, is a potent inhibitor of ferroptosis (87). Treatment with glycyrrhizin, an HMGB1 inhibitor, could increase GSH and GPX4 levels, and activate the Nrf2/HO-1/HMGB1 pathway, blocking ferroptosis in ALF models induced by the co-injection of d-galactosamine (d-GalN) and lipopolysaccharides (LPS) (88). Researchers have found that promethazine, one of the cytochrome P450 substrate compounds, functions as a lipid peroxyl radical scavenger to inhibit ferroptosis, ameliorating LPS/GalN-induced ALF and decreasing cell death (89). APAP overdose induced VDAC1 oligomerization in hepatocellular cells, and thus the application of VDAC1 oligomerization inhibitor VBIT-12 alleviated ferroptosis by protecting mitochondria and restoring ceramide and cardiolipin levels (90). In CCl4-induced acute liver injury, treatment with chloroquine downregulated HMGB1 levels and NF-kB expression, and increased the Bax/Bcl-2 ratio and caspase-3 activation in hepatic tissue, demonstrating inhibition of HMGB1-mediated inflammation and activation of pro-apoptotic pathways (91).

Cell-based therapy is a fast-developing research field in the restoration of liver function and treatment of various liver diseases and acts by remodeling and repairing the liver injury niche and promoting parenchyma regeneration. Hepatocyte transplantation has been explored for specific metabolic liver disorders, especially for pediatric patients and liver failure conditions (92), as well as for various drug-induced ALFs such as halothane, dilantin, and multiple polysubstance misuses (93, 94). Apart from human primary hepatocyte transplantation as the first form of cell therapy, stem cells are considered very promising for liver injury as well, such as embryonic stem cells (ESCs), hematopoietic stem cells (HSCs), induced pluripotent stem cells (iPSCs), and mesenchymal stem cells (MSCs), which have the ability to self-renew and differentiate, or function in immunomodulation, fibrosis degradation, and hepatocyte proliferation (95–97). Transplantation of ESC-derived hepatocytes into mice with APAP-induced acute liver failure rescued hepatic function (98). The infusion of umbilical cord-derived CD362-positive MSCs was demonstrated to alleviate hepatic inflammation by expanding the anti-inflammatory M2 phenotype of macrophages in a primary sclerosing cholangitis mouse model (99). Transplantation of human bone marrow mesenchymal stem cells (hBMSCs) into immunodeficient mice with fulminant hepatic failure induced restoration of the damaged liver by enhancing hBMSCs differentiation into cholangiocyte *via* delta ligand-like 4 (DLL4) activation (100). The effect of induced hepatocyte-like cells (iHEPs) generated by direct reprogramming from mouse embryonic fibroblast on acute liver injury was investigated in a CCl_4_-induced mouse model and significantly attenuated acute liver injury (101). Meanwhile, clinical trials on hepatocyte or stem cells transplantation have been investigated or are ongoing, among which MSCs are considered the most promising candidates (95). The overall safety and effectiveness of MSC therapy was evaluated and confirmed in 14 pediatric patients with urea cycle disorders (UCDs) or Crigler-Najjar (CN) syndrome 6 months post transplantation (102). A clinical trial on autologous MSCs transplantation for 158 patients, including teenagers, with liver failure caused by hepatitis B has been carried out to assess the short-term therapeutic effects and long-term fatality (NCT00956891) (**Supplementary Table 1**).

Additionally, various immune cells such as macrophages, Treg, monocytes, and DCs, are highlighted in the development of therapies for liver disorders. Given that macrophages play a crucial role in the progression and resolution of ALI, the value of bone marrow-derived macrophages (BMDMs) was identified as a cell-based therapy in an APAP-ALI mouse model. Injection of activated BMDMs reduced hepatocellular necrosis, infiltrating neutrophils, circulating proinflammatory cytokines levels, and HMGB1 translocation, meanwhile promoting hepatocyte and endothelium proliferation (103). Treg, which is pivotal in preventing autoimmunity, alloimmunity, and maintaining immunological tolerance and tissue homeostasis, has attracted great attention in adoptive cell therapy (104–106). The safety of autologous Treg infusion for four patients with autoimmune hepatitis (AIH) was evaluated, indicating that approximately 22-44% of infused Tregs homed to and resided in the liver for up to 72 hours, and acted to suppress the tissue-damaging effector T cells (107). In animal models, Treg could ameliorate APAP-induced immune-mediated liver injury and also inhibit CCl_4_ or triptolide-induced liver injury (51, 108–110). Treg played a crucial role in the MSC-based alleviation of acute liver inflammation, and adoptive transfer of MSC-primed Tregs completely attenuated α-GalCer-induced ALF, which indicated a novel therapeutic approach in Treg-based therapy of ALF (111).

## 6 Summary

In this review, we have focused on the immune activation and regulation in pediatric acute liver injury in the SARS-CoV-2 pandemic era. We summarized both the cellular and molecular crosstalk between immune cells and liver cells in this process as well as therapeutic targets and cell therapy strategies. Considerable progress in multiple therapeutic approaches has been made in the setting of acute liver injury and failure, especially in mice models. Based on former results, the combination of different therapeutic approaches might be promising for cell-based therapy of acute liver injury. However, more clinical trials are still required for validation of their safety and efficacy.

## Author contributions

All authors contributed to the article and approved the submitted version.
