# Supplementary material for: Orchestrated regulation of immune inflammation with cell therapy in pediatric acute liver injury
Source: Front Immunol. 2023 Jun 22;14:1194588. doi: 10.3389/fimmu.2023.1194588 (PMC10323196; doi:10.3389/fimmu.2023.1194588)
Supplement: Supplementary file 1 [file Table_1.docx]

**Table 1: Clinical trials of cell therapy for patients with liver failure including pediatric patients.**

| **ClinicalTrials.gov Identifier** | **Recruitment Status** | **Title** | **Study Design** | **Intervention** | **Enrolment** | **Condition** |
| --- | --- | --- | --- | --- | --- | --- |
| NCT01724398 | Recruiting | Umbilical Cord Mesenchymal Stem Cells Transplantation Combined With Plasma Exchange for Patients With Liver Failure | Randomized Parallel  PhaseⅠ/Phase Ⅱ | umbilical cord mesenchymal stem cells transplantation by peripheral vein slowly for 30minutes. (1×10^5^/Kg, once a week, 4 times) | 120 | Liver Failure |
| NCT00956891 | Completed | Therapeutic Effects of Liver Failure Patients Caused by Chronic Hepatitis B After Autologous MSCs Transplantation | Case-Control Retrospective | autologous MSCs transplantation plus medical treatments | 158 | Liver Failure  Liver failure patients with HBV infection |
| NCT01221454 | Active, not recruiting | Efficacy of Allogenic Bone Marrow Stem Cells Transplantation in Patients With Liver Failure Resulting From Chronic Hepatitis B | Non-Randomized Parallel Assignment  Phase Ⅱ | 30ml allogenic bone marrow stem cells were infused to patients using interventional method via portal vein or hepatic artery as well as conserved therapy | 60 | Liver Failure |
| NCT01322906 | Active, not recruiting | Allogeneic Bone Marrow Mesenchymal Stem Cells Transplantation in Patients With Liver Failure Caused by HBV | Randomized Parallel  PhaseⅠ/Phase Ⅱ | allogeneic bone marrow mesenchymal stem cells transplantation by peripheral vein (Group 1, 2×10^5^/Kg, once a week, 4 times; Group 2.1×10^6^/Kg, once a week, 4 times;  Group 3.5×10^6^/Kg, once a week, 4 times). | 120 | Liver Failure |
| NCT03863002 | Not yet recruiting | Safety and Efficacy of Mesenchymal Stem Cell Transplantation for Acute-on-Chronic Liver Failure | Randomized Parallel  PhaseⅠ/Phase Ⅱ | Mesenchymal stem cell transplantation via peripheral vein: 1.0-10×10^5^ MSCs/kg body weight administered via peripheral vein at week 0, 1, 2, 3 weeks |  | Liver Failure, Acute on Chronic |
